# Are Single Doctors More Focussed in Career Progression?

**DOI:** 10.1192/bjo.2022.106

**Published:** 2022-06-20

**Authors:** Ramy Metwali, Divya Vikraman Chandrika, Siddhartha Baiju

**Affiliations:** 1 NHS Wales, Cardiff, United Kingdom; 2 NHS Wales, PONT-Y-CLUN, United Kingdom; 3 NHS Wales, Bridgend, United Kingdom

## Abstract

**Aims:**

It is commonly assumed that single doctors are more focused on career progression than doctors in a relationship/ or having dependents. In this study, we tried to find out if it's true. We measured this among doctors in Psychiatry (from training – to consultants’ level) across Wales.

**Methods:**

We designed an anonymized online survey and distributed it among Psychiatry doctors.

We categorized the relationship status as

- Single
- In a relationship – committed, engaged, married, living together
- Separated, Divorced, Widowed
- Others

We measured the career progression on the basis of the following criteria:

1. Why they were interested in Psychiatry
2. Number of years taken to complete or intending to complete training.
3. Having met the portfolio criteria for their level.
4. Undertaking RCPsych Exams
5. Having missed any opportunities related to career.

We analysed the data from the survey using an online tool.

The survey included questions that measured the link between career progression/choices and relationship Status/responsibilities. We used the Likert Scale, yes or no questions, and free text boxes.

**Results:**

We received 66 responses and we divided them into three groups according to their relationship status which are the single group, relationship group, Divorced/ Separated/ Widowed group, and compared the results between them. We got some interesting results as follows.

- Single participants seem to be less limited in choosing the specialty, Exam Preparation, and overall career progression.
- A significant proportion of people in relationships felt limited in choosing the Specialty and workplace. Had less time for Exam preparation and have missed career opportunities.
- Females in Relationships were more restricted.
- The divorced / Separated / Widowed group did not feel limited, however, affected their exams, and have missed opportunities.

**Conclusion:**

Single doctors seems to have overall better opportunities in career progression compared to people in relationship. However, our sample size was small especially in single group. A bigger study is needed to conclude the impact of relationship in career progression.

